# Effects of 14-days bismuth- and tetracycline-containing quadruple therapy with concomitant regimen for the first line *Helicobacter* *pylori *eradication

**DOI:** 10.22088/cjim.14.4.676

**Published:** 2023

**Authors:** Arash Kazemi Veysari, Ali Rahimi, Iradj Maleki, Hafez Tirgar Fakheri, Tarang Taghvaei, Vahid Hosseini, Seyed Mohammad Valizadeh Toosi, Danial Masoumi, Zohreh Bari

**Affiliations:** 1 Department of Internal Medicine, Gut and Liver Research Center, Non-Communicable Disease Research Institute, Mazandaran University of Medical Sciences, Sari, Iran; 2 Mazandaran University of Medical Sciences, Sari, Iran; 3 Student of Internal Medicine, Mazandaran University of Medical Sciences, Sari, Iran

**Keywords:** *Helicobacter pylori*, Bismuth, Tetracycline, Clarithromycin

## Abstract

**Background:**

*Helicobacter pylori* (*H. pylori*) has infected about 50% of the world’s population and it is the main cause for peptic ulcer, gastric adenocarcinoma and even a major cause for gastric MALT lymphoma.

**Methods:**

This study was performed in Mazandaran, Sari, situated in North of Iran. Three-hundred and twenty-eight adult patients with endoscopically approved gastric or duodenal ulcers or erosions and *H. pylori* infection were randomly divided into 2 groups to receive either 14 days PABT (Pantoprazole 40 mg, Amoxicillin 1 g, Bismuth 425 mg (all twice daily) and Tetracycline 500 mg four times a day) and PACM (Pantoprazole 40 mg, Amoxicillin 1g, Clarithromycin 500 mg, and Metronidazole 500 mg, all twice daily). To evaluate *H. pylori *eradication, fecal *H. pylori *antigen test was performed 8 weeks after treatment.

**Results:**

The eradication rates were 94.51% in the PABT and 91.46% in PACM group based on the intention to treat analysis. Moreover, the eradication rates were 95.58% and 92.72% according to per-protocol analysis, respectively. Also, both groups had very low rates of severe side effects.

**Conclusion:**

Regarding the ideal eradication rates achieved by both treatment groups and the low rates of severe side effects, both treatment protocols can be prescribed for H. pylori eradication in North of Iran.

*Helicobacter pylori* (*H. pylori*) has infected about 50% of the world’s population (1). Therefore, *H. pylori *infection is a global health issue. It is the main cause for peptic ulcer, gastric adenocarcinoma and even a major cause for gastric MALT lymphoma (1, 2). Despite more than 30 years of trying to cure *H. pylori *infection, the ideal treatment regimen that can eradicate the organism is still not achieved in many geographic regions. (3-5). In many clinical trials and meta-analyses, treatment failure rates of up to 20% have been reported (6-8). This indicates the need to introduce new treatment regimens. Several treatment protocols have been evaluated for *H. pylori* eradication, including the standard triple therapy of 7, 10 and 14 days, the Bismuth-based quadruple therapy, 10- and 14-day concomitant regimens, 10- and 14-day sequential regimens, and 10, 12 and 14-day hybrid therapies. In all of these treatment regimens, the main goal is to achieve more than 85% eradication rate with few side effects. In areas where antibiotic resistance to Clarithromycin is high (more than 15%), standard triple therapy should be avoided unless an antibiogram is performed. In these areas, it is recommended to use 14-days Bismuth-based quadruple therapy or concomitant regimen (9, 10). Recent studies from Iran have shown that concomitant therapy can be an acceptable regimen for H. pylori eradication (11). On the other hand, tetracycline-containing regimens have been rarely investigated in Iranian studies and the publications are not new.

Therefore, their results cannot demonstrate the present negative effects of antibiotic resistance on *H.* *pylori *treatment protocols (12). During previous years, the rate of H. *pylori *antibiotic resistance has significantly increased (13-17). Therefore, we designed a study to evaluate and compare the efficacies of Bismuth- and Tetracycline-based quadruple therapy with 14-days concomitant regimen.

## Methods

**Study** **design: **This was a double-blind, randomized clinical trial, performed for *H. pylori* treatment in Sari, situated in Mazandaran province, in North of Iran; from August 2018 to August 2020. The study was conducted after approval by the Ethics Committee of Mazandaran University of Medical Sciences (IR.MAZUMS.IMAMHOSPITAL.REC.1398.045). Patients were enrolled after signing informed consent. 

**Participants: **Adult patients more than 18 years old (including both men and women) with endoscopically approved gastric or duodenal ulcers or erosions and *H. pylori* infection who had not received previous *H. pylori* treatment enrolled this study. The presence of *H. pylori* infection was confirmed by rapid urease test and histopathologic evaluation(18). The exclusion criteria were as follows: breast-feeding, pregnancy, previous gastric surgery, concomitant use of some drugs including anticoagulants, corticosteroids and ketoconazole, history of ischemic heart disease, lung diseases, chronic renal failure, liver disease, any kind of malignancy and history of allergy to the antibiotics used in each protocol.

**Interventions: **Patients who were found eligible for this study were randomly assigned to two treatment groups. Demographic information (including age and gender), history of gastrointestinal bleeding, taking non-steroidal anti-inflammatory drug (NSAID) and endoscopic findings were recorded. In group A (PABT), patients received Pantoprazole 40 mg, Amoxicillin 1 g, Bismuth 425 mg (all twice daily) and Tetracycline 500 mg QID. Group B received (PACM) Pantoprazole 40 mg, Amoxicillin 1g, Clarithromycin 500 mg, and Metronidazole 500 mg (all twice daily). The duration of both protocols was 14 days. 

**Outcomes: **After the treatment courses were completed, patients were evaluated to assess possible side effects and the level of compliance with the treatments. Patients were also asked to inform the physician by call in case of severe adverse effects during the 2 weeks of receiving treatment drugs. The severity of adverse effects was classified based on their impact on daily activities: mild (no interference with daily activities), moderate (slightly affecting regular activities), and severe (avoiding daily activities). Drug compliance was classified according to the duration of taking the medications in comparison to the complete treatment duration: excellent (more than 90%), good (70-90%), and poor (less than 70%). To evaluate *H. pylori *eradication, fecal *H. pylori *antigen test was performed 8 weeks after treatment.

**Statistical analysis** **: **Data were analyzed using t-test and chi-square test by SPSS software (Version 18), as appropriate. Eradication rates were calculated according to intention to treat (by including all participants in the analyses) and per-protocol analysis (by including only patients who completed the whole protocol and had excellent compliance to treatment). Also, *p*-values less than 0.05 were considered statistically significant.

## Results

In total, 350 patients were assessed for eligibility. Twelve patients who did not meet the inclusion criteria (had previously received eradication therapy) were excluded. Ten patients declined to participate. Finally, 328 patients were randomly assigned to PABT and PACM treatment groups (164 in each group. Demographic characteristics of the patients are shown in table 1. There was no significant difference between baseline demographic characteristics of the two groups (*p*>0.05).

**Table 1:** Baseline demographic characteristics of the patients in both groups

**Variables**	**Total** **(n=328)**	**PABT** **(n= 64)**	**PACM** **(n= 64)**	**P-value**
**Age (year)**	46.31 ± 13.7	46.4 ± 12.21	46.25 ± 14.6	0.894
**Male sex (%)**	142 (43.2)	63 (38.4)	79 (48.1)	0.058
**Smoker (%)**	41 (12.5)	23 (14.02)	18 (10.97)	0.404
**History of taking non-steroidal anti-inflammatory drugs (NSAIDs) (%)**	49 (14.9)	21 (12.80)	28 (16.97)	0.278
**History of gastrointestinal bleeding (GIB) (%)**	9 (2.7)	4 (2.44)	5 (3.05)	0.735

PABT: Pantoprazole 40 mg, Amoxicillin 1 g, Bismuth 425 mg (all twice daily) and Tetracycline 500 mg four times a day

PACM: Pantoprazole 40 mg, Amoxicillin 1g, Clarithromycin 500 mg, and Metronidazole 500 mg, all twice daily

All patients completed the protocol and performed fecal *H. pylori* antigen test. According to intention to treat analysis, eradication rates were 94.51% in the PATB and 91.46% in PACM group. However, per-protocol eradication rates were calculated regarding compliance to treatment. Accordingly, per-protocol eradication rates were 95.58% and 92.72% in the mentioned groups, respectively.The eradication rates were not statistically different between the two groups either by per-protocol or intention to treat analyses. The frequency and types of side effects of therapies are shown in table 2. The most frequent adverse effects of treatment were bitter taste (in PACM group) and epigastric pain (in PATB group). However, they were mostly mild. Regarding the severity of side effects, no statistically significant difference was observed between the two groups (P = 0.640, table 2).

 Regarding compliance to treatment, 82.9% of the patients in the PATB and 67.07% of the patients in the PACM group had used more than 90% of their medications. The most common reason for drug withdrawal was the occurrence of side effects, although most of the side effects were not severe. 

**Table 2:** Frequency and severity of side effects; and compliance to treatment in both groups

**Variable**		**PABT**	**PACM**	**P-value**
**Side effect**	Bitter taste	2	48	0.060
	Epigastric pain	16	2	
	Weakness	14	0	
	Constipation	4	7	
	Nausea and vomiting	0	7	
	Diarrhea	2	5	
	Itching	0	7	
	Headache	3	3	
	Vertigo	0	5	
	Glossitis	0	3	
	Rash	2	0	
	Decreased appetite	2	0	
	Fever	0	2	
	Urine discoloration	1	0	
**Severity of Side effect**	Mild	45	53	0.640
	Moderate	1	7	
	Severe	0	2	
**Treatment Compliance**	Excellent	136	110	0.004
	Good	23	42	
	Poor	5	12	

PABT: Pantoprazole 40 mg, Amoxicillin 1 g, Bismuth 425 mg (all twice daily) and Tetracycline 500 mg four times a day

PACM: Pantoprazole 40 mg, Amoxicillin 1g, Clarithromycin 500 mg, and Metronidazole 500 mg, all twice daily

## Discussion

*Helicobacter pylori* is strongly associated with peptic ulcer disease and gastric cancers. Therefore, its eradication is recommended in all patients with peptic ulcer. However, its treatment requires the administration of at least two antimicrobial drugs. Antibacterial drugs most commonly used to eradicate *H.* *pylori *infection include Metronidazole, Amoxicillin, Bismuth compounds, and Tetracycline (19, 20). Different treatment regimens with different efficacy and side effects have been proposed to eradicate *H.* *pylori*. The optimal effectiveness of three- or four-drug regimens in European and Western countries is 85 to 95%, but due to the high level of resistance to antibiotics in Iran, the rate of eradication is usually not optimal (20-23).

 In our study, the success rates achieved by both protocols were over 90%. Also, although, the eradication rate of *H.* *pylori *was higher in PATB treatment group, however, no significant difference was observed between the eradication rates achieved by the two groups. Since the ideal regimen for *H.* *pylori* eradication should eradicate the organism in more than 85% to 90% of the cases (20, 22). Therefore, both treatment regimens used in this study were adequately effective. In European and Western countries, increased resistance to Clarithromycin due to the widespread use of this antibiotic in children and adults has increased the rate of failure in *Helicobacter pylori* treatment by triple therapies. However, European guidelines for the treatment of H. *pylori *suggests triple treatment with PPI, Amoxicillin and Clarithromycin (or Metronidazole) for 14 days or quadruple treatment with PPI, Amoxicillin, Clarithromycin and Metronidazole for 10-14 days (24-26, 7). 

In a previous review article by Fakheri et al., quadruple therapy consisted of a PPI, Metronidazole, Bismuth and Tetracycline was reported as a suitable option for *pylori* eradication in West Asia (27).The results of the present study are in concordance with the mentioned review article. Also, Hsu et al. reported 96% *H.* *pylori* eradication rate by 14-days Bismuth- and Tetracycline-containing quadruple therapy in Thailand (28). Furthermore, in 2018, Huang et al. reported 90% H. *pylori* eradication rate by 14-days Tetracycline-containing quadruple therapy as the third line eradication regimen (29).

 Their eradication success rate was in accordance with our eradication rate. Regarding adverse reactions, the ideal rate of adverse effects caused by H. *pylori* treatment drugs must be lower than 5%. In our study, the most common complications observed in the PATB treatment group were epigastric pain (9.7%) and weakness (8.5%), respectively. In PACM group, mouth bitterness was the most common side effect (29%). However, no statistically significant difference was observed between the two study groups regarding total side effects (P = 0.060). Furthermore, although the total rates of side effects were significantly high in both groups (28% in PATB group and 37.8% in PACM group, respectively), the rates of severe side effects were very low. Only 2 patients from PACM group (1.2%) reported severe side effects and the severity of most side effects were mild. 

In conclusion, both Bismuth- and Tetracycline-based quadruple therapy and 14-days concomitant regimen could achieve more than 90% *H.* pylori eradication rates. Regarding their ideal eradication rates and the low rates of severe side effects, both regimens seem to be suitable options to be prescribed for first-line *H.* pylori treatment in this geographic region.
